# Evaluation of the portability of computable phenotypes with natural language processing in the eMERGE network

**DOI:** 10.1038/s41598-023-27481-y

**Published:** 2023-02-03

**Authors:** Jennifer A. Pacheco, Luke V. Rasmussen, Ken Wiley, Thomas Nate Person, David J. Cronkite, Sunghwan Sohn, Shawn Murphy, Justin H. Gundelach, Vivian Gainer, Victor M. Castro, Cong Liu, Frank Mentch, Todd Lingren, Agnes S. Sundaresan, Garrett Eickelberg, Valerie Willis, Al’ona Furmanchuk, Roshan Patel, David S. Carrell, Yu Deng, Nephi Walton, Benjamin A. Satterfield, Iftikhar J. Kullo, Ozan Dikilitas, Joshua C. Smith, Josh F. Peterson, Ning Shang, Krzysztof Kiryluk, Yizhao Ni, Yikuan Li, Girish N. Nadkarni, Elisabeth A. Rosenthal, Theresa L. Walunas, Marc S. Williams, Elizabeth W. Karlson, Jodell E. Linder, Yuan Luo, Chunhua Weng, WeiQi Wei

**Affiliations:** 1 Northwestern University, Evanston, USA; 2 National Human Genome Research Institute, Bethesda, USA; 3 Pennsylvania State University, Hershey, USA; 4 Kaiser Permanente Washington Health Research Institute, Seattle, USA; 5 Mayo Clinic, Rochester, USA; 6 Massachusetts General Hospital, Boston, USA; 7 Mass General Brigham, Somerville, USA; 8 Columbia University, New York, USA; 9 Children’s Hospital of Philadelphia, Philadelphia, USA; 10 Cincinnati Children’s Hospital Medical Center, Cincinnati, USA; 11 Geisinger, Danville, USA; 12 Intermountain Healthcare, Salt Lake City, USA; 13 Vanderbilt University Medical Center, Nashville, USA; 14 Icahn School of Medicine at Mount Sinai, New York, USA; 15 University of Washington, Seattle, USA; 16 Brigham and Women’s Hospital, Boston, USA

**Keywords:** Translational research, Genetics research, Communication and replication, Computational models, Data integration, Data mining, Data processing, Machine learning, Programming language, Software, Standards

## Abstract

The electronic Medical Records and Genomics (eMERGE) Network assessed the feasibility of deploying portable phenotype rule-based algorithms with natural language processing (NLP) components added to improve performance of existing algorithms using electronic health records (EHRs). Based on scientific merit and predicted difficulty, eMERGE selected six existing phenotypes to enhance with NLP. We assessed performance, portability, and ease of use. We summarized lessons learned by: (1) challenges; (2) best practices to address challenges based on existing evidence and/or eMERGE experience; and (3) opportunities for future research. Adding NLP resulted in improved, or the same, precision and/or recall for all but one algorithm. Portability, phenotyping workflow/process, and technology were major themes. With NLP, development and validation took longer. Besides portability of NLP technology and algorithm replicability, factors to ensure success include privacy protection, technical infrastructure setup, intellectual property agreement, and efficient communication. Workflow improvements can improve communication and reduce implementation time. NLP performance varied mainly due to clinical document heterogeneity; therefore, we suggest using semi-structured notes, comprehensive documentation, and customization options. NLP portability is possible with improved phenotype algorithm performance, but careful planning and architecture of the algorithms is essential to support local customizations.

## Introduction

Accurate extraction of complete and detailed phenotypic information from large-scale electronic health record (EHR) data improves efficiency and accuracy of precision medicine research. However, structured data alone is often insufficient to fully identify or describe many conditions, particularly when an attribute is not commonly billed for or requires nuanced interpretation^1–4^. Natural language processing (NLP) and machine learning (ML) promise to enable deep phenotyping using nuanced EHR narratives^5–8^.

Both sophisticated NLP pipelines, such as MedLEE^9^, CLAMP^10^, cTAKES^11^ and MetaMap^12,13^; and simpler rule-based approaches combining regular expressions (RegEx) and logic; have increasingly been leveraged for deep phenotyping^14^. However, it is challenging to achieve broad generalizability and phenotype algorithm portability given the disparate EHR systems and heterogeneous documentation approaches used by clinicians^15^. For instance, Sohn et al. reported how variations in asthma related clinical documentation between two cohorts affect NLP system portability^16^. Additionally, document types and structures vary among EHRs, and some sites have more unstructured data than others. Abbreviations, terminologies, and other language usage also varies across sites, clinicians, and time. For example, Adekkanattu et al. reported variability in system performance due to the heterogeneity of local text formats and lexical terms used to document various concepts, across three different institutions assessing the portability of a specialized echocardiography information extraction system^17^.

The biomedical NLP community has developed a number of approaches to address these issues, including measuring semantic similarity of text, deploying ensemble NLP systems, using comprehensive term dictionaries, and converting text into data standards, such as Fast Health Interoperability Resources (FHIR) and the Observational Medical Outcomes Partnership (OMOP) common data model (CDM)^18^. Specifically, Liu et al.^19^ demonstrated that ensembles of NLP systems can improve portability through both generic phenotypic concept recognition and patient specific phenotypic concept identification over individual systems. Furthermore, Jiang et al. leveraged the FHIR standard to develop a scalable data normalization pipeline that integrates both structured and unstructured clinical data for phenotyping^20^. Lastly, Sharma et al. developed a portable NLP system by extracting phenotype concepts, normalizing them using Unified Medical Language System (UMLS), and mapping them to the OMOP CDM^21^.

The eMERGE (electronic MEdical Records and GEnomics) Network was organized and funded by the National Human Genomic Research Institute (NHGRI) in 2007 to study the intersection of genomics and EHRs^22–26^. One of the network’s most enduring contributions is the development of computable phenotypes to identify common diseases within EHRs for genetic research. Each phenotype algorithm is validated across multiple sites and is publicly available in the Phenotype KnowledgeBase (PheKB.org)^27^. Over the past fourteen years, the eMERGE Network has accumulated considerable experience in phenotyping algorithm development, validation, and implementation^17,22–25,28–32^. This collaboration among multiple participating institutions provides rare opportunities to explore NLP performance and portability for the ‘big data’ in EHRs across diverse settings. An ongoing critical task remains identification of the knowledge gap of best practices in development, validation, and implementation of portable phenotype algorithms using NLP.

## Objective

One of the goals of phase III of the eMERGE Network (2015–2020) was to incorporate NLP/ML into existing eMERGE phenotype algorithms to improve their performance and/or better ascertain sub-phenotypes. To that end, in 2019–2020, a 1 year pilot study was conducted to test the feasibility of deploying portable phenotype algorithms that incorporated NLP components into existing rules-based phenotype algorithms. Specifically, we aimed to use NLP to identify sub-populations and improve existing phenotype algorithms. As we are identifying cases (and also sometimes controls) for genetic research, having the highest number possible of accurately identified patients (cases) with the given phenotype is important. Thus, improvement was defined as either improved recall, to increase the number of cases; and/or improved precision to correctly identify a higher percentage of true cases. We hypothesized that development of portable, accurate, and efficient NLP tools for multi-site application depends on the availability of intra- and inter-site human and technological resources, due to highly variable experience in the field, including among our sites. These must be capable of exposing and addressing the various sources of heterogeneity, such as different environments, which impact an NLP system’s ability to accurately extract information. Reflecting on this eMERGE work, the objective of this paper is to: (1) report challenges we faced during implementation of eMERGE phenotype algorithms with NLP/ML- components added and, (2) recommend best practices we encountered and/or found upon review, to help others overcome those challenges, in order to implement portable phenotype algorithms, especially those with NLP/ML components.

## Materials and methods

In order to achieve these objectives, an NLP sub-workgroup of the eMERGE Phenotyping Workgroup was formed that included representatives from nine eMERGE sites: Children’s Hospital of Philadelphia (CHOP), Cincinnati Children’s Hospital Medical Center (CCHMC), Columbia University, Geisinger, Harvard/Mass General Brigham, Kaiser Permanente Washington and the University of Washington (KPWA/UW), Mayo Clinic, Northwestern University (NU), and Vanderbilt University Medical Center (VUMC). Based on scientific merit and predicted difficulty, the group selected six phenotypes with existing computable phenotype algorithms to enhance with NLP: chronic rhinosinusitis (CRS)^33^, electrocardiogram (ECG) traits^34^, systemic lupus erythematosus (SLE)^35^, asthma/chronic obstructive pulmonary disease (COPD) overlap (ACO)^36^, familial hypercholesterolemia (FH)^37^, and atopic dermatitis (AD)^38^. All of the algorithms were case – control algorithms; specifically, cases were patients with, and controls without, the phenotype, as defined by each algorithm. Sub-phenotypes included traits on ECG reports such as Brugada syndrome, CRS with and without nasal polyps, and sub-types of SLE and AD.

To reduce study heterogeneity to accommodate time limitations and to lower barriers to implementation by clinicians with minimal NLP training, we restricted NLP pipelines to those with which we had experience^39–44^, and that were a reasonable reflection of the variety of NLP tools currently used in healthcare settings, as seen in a recent review^45^. To this end, NLP platform selection was based on a survey of platforms that sites had the most experience using. The selected tools were: cTAKES^11^, MetaMap^12,13^, and/or regular expressions (RegEx), along with two commonly adopted negation detection modules: NegEx and ConText^46,47^, which are rule-based. The modified AD and COPD/ACO phenotype algorithms also had ML components, for which custom code written in Python and Java was used, respectively. The phenotypes, along with goals and selected tools, are shown in Table 1, and more details of the algorithms are available on PheKB.org^27^.

**Table 1:** Phenotype goals & NLP tool selection.

Phenotype	Lead	Validation	Goal	Tool(s)
Chronic rhinosinusitis	Geisinger	NU	Improve precision	cTAKES
ECG traits	VUMC	Mayo clinic	Enrich phenotype & extract sub-phenotypes	RegEx
Systemic lupus erythematosus	NU	VUMC	Improve sensitivity & extract sub-phenotypes	MetaMap w/RegEx
Asthma/chronic obstructive pulmonary disease overlap	Harvard	KPWA/UW	Improve sensitivity	RegEx, Java
Familial hypercholesterolemia	Mayo clinic	Geisinger	Improve precision	cTAKES
Atopic dermatitis	NU	CHOP, Marshfield clinic, Geisinger	Improve sensitivity (for adults) & extract sub-phenotypes	cTAKES, RegEx, Python

*NU* Northwestern University, *VUMC* Vanderbilt University Medical Center, *KPWA/UW* Kaiser Permanente Washington and the University of Washington, *CHOP* The Children’s Hospital of Philadelphia.

To validate the phenotype algorithms according to our objectives, we focused on validating if patients were correctly identified as cases (and/or controls) by both the original and new NLP-enhanced phenotype algorithms. The original algorithms were previously validated^33–38^. Then for this study, the “lead” (primary) site added NLP component(s) to the original algorithm, which they had previously led (with one exception, AD, which was previously led by a pediatric site, but in this pilot project was led by a site focused on adults). Then the lead site validated the NLP/ML-enhanced phenotype algorithm, via manual chart review of randomly selected subsets of: patients’ charts, and as needed, clinical notes for those patients. Next, as is typical in the development of eMERGE phenotype algorithms^23^, the lead site worked with at least one “validation” (secondary) site to further adjust the algorithms as needed, until satisfactory precision and recall was achieved, calculated via the manual reviews. Specifically, the eMERGE network’s phenotype algorithm validation procedures^23^, which were used here, involve sites having clinicians experienced in diagnosing and treating the given phenotype, or medical professionals who are highly trained, to ascertain presence or absence of the phenotype in the entire patient health record (not just the clinical text), and if necessary its detailed characteristics, such as signs and symptoms. As also is typical within eMERGE, if possible, at least 2 people reviewed the charts and also reviewed at least a few of the same charts in the beginning to ensure inter-rater reliability, while a more senior person adjudicates any differences where possible; or, if there is only a single reviewer, the person is an expert for the phenotype. For example, for the ACO phenotype development, 2 pulmonologists reviewed and a 3rd pulmonologist reconciled discordant labels; while at KPWA for the same phenotype, chart reviews were conducted by one professional non-clinician chart abstractor with access to an MD clinician to assist the abstractor in resolving any questions/concerns that were beyond the abstractors’ competency. Similarly, at Mayo and Geisinger, a single MD reviewed the charts and at VUMC, a senior cardiologist reviewed all ECG reports and for SLE, a rheumatologist doing SLE research did that review. A lead site reviews approximately 50 patients’ charts and at least one validation (secondary) site subsequently reviews approximately 25 charts: the number of charts reviewed is sometimes higher depending on the phenotype^23–27^, which did occur in this study. If the phenotype algorithm is for identifying both cases and controls, then the total number of charts reviewed includes both (for example, approximately 25 potential cases and 25 potential controls when the total is 50 charts reviewed)^23–27^, as seen for multiple phenotypes in this study. Finally, the phenotype algorithms were disseminated to all participating sites for implementation, and further iteratively improved as needed based on feedback from implementing sites. Final accuracy statistics were re-calculated, if necessary, after all modifications were made for reporting here.

We then retrospectively compared NLP methods and tools to assess performance, portability, and ease of use. To do this, we asked sites to report their lessons learned for creating and sharing NLP/ML algorithms via a brief informal survey about each phenotype algorithm they developed, validated, and/or implemented (questions asked are listed in Supplementary Appendix A). Quantitatively, sites were asked to report performance (especially recall and precision) at both the lead and (secondary) validation sites, for both the original and modified (NLP added) phenotype algorithms. Sites were also asked to estimate the amount of resources and time it took to complete development, validation, and implementation. These estimates were based on approximations after the work was completed. In addition, since personnel typically did not spend 100% of their time on the algorithms, time estimates are variable as they are dependent on proportion of effort. Also, some sites optionally separated the expertise of people needed to complete the task (e.g. clinical, informatics, and EHR analysts). Physical resources were reported as the number of servers needed to query the data and/or execute the algorithms. Qualitatively, sites were asked to report on how difficult they felt each algorithm was to implement; how portable it was, including any local customizations that were needed for the algorithm to perform; and any other issues identified by sites when sharing, including technical or performance issues. Additional qualitative feedback on the experience was informally collected at monthly workgroup meetings and from direct emails from sites.

Using grounded theory^48^, a thematic analysis was conducted by two authors (JAP, LVR) via independent review of all qualitative feedback. First, open and axial coding on categories of issues or concerns was completed to identify key phrases and loosely categorize them. The coders used selective coding to refine axial codes into a comprehensive hierarchical codebook, independently re-coded the feedback, and reviewed to achieve consensus. Emergent themes were identified through iterative review of the codes. Next, we prepared a review and summary of lessons learned, including (a) challenges for each theme; (b) corresponding best practices to address those challenges based on existing published evidence and/or experience of the eMERGE Network; and (c) if applicable, opportunities for future research. Finally, to assess credibility, the results were presented to co-authors, then lessons and recommendations were further refined as needed.

### Ethics, consent and permissions

Informed consent was obtained from all subjects involved in the study per each site’s Institutional Review Board (IRB). The research was performed in accordance with the relevant guidelines and regulations for use of human participants’ biomedical data, including those of each site’s approved IRB protocols, and in accordance with the Declaration of Helsinki.

## Results

For each phenotype algorithm, Table 2 presents accuracy statistics and personnel required. Although not reported by all sites, the roles of personnel involved included programmers, clinicians, and computational linguists. Although most sites reviewed 50–100 patients’ charts as is standard within eMERGE for validation of phenotype algorithms, the range did vary: lead sites reviewed anywhere from 46 to 972 charts, with a median of 100 charts reviewed, and validating sites reviewed 50–950 charts, with a median of 65 charts reviewed. From those patient chart reviews, for all but one algorithm (SLE, where overall the precision decreased), the precision and recall overall were unchanged or improved at both the lead (primary) and (secondary) validation sites. Changes in accuracy statistics for sub-phenotypes varied between sub-phenotypes and developing and validating sites. Differences in phenotype algorithm performance were not associated with the tools used. Only two sites noted the number of records in the EHR (containing both clinical text and discrete data such as labs) that were used, and for implementation of the final NLP/ML enhanced phenotype algorithm: for the ECG algorithm, it was noted that just over 1 million ECG records from the EHR were used in VUMC’s implementation; for the SLE algorithm, 185,838 notes were processed from 4468 patients for VUMC’s implementation; and for the AD algorithm, 4094 patients’ notes, labs, and/or codes were reviewed for another site’s implementation.

**Table 2:** Summary of the NLP/ML component outcomes.

Phenotype	People Involved	Charts reviewed	Precision	Recall	Comments
Chronic rhinosinusitis	2	126	76% → 78–83%	97% → 100%	Also significant improvement on specificity
ECG traits	1–3	1050	Cases: 80–100% Controls: 94–99%	N/A	Unable to extract 1 sub-phenotype; precision varied between sub-phenotypes
Systemic lupus erythematosus	2–3	1022	99% → 96%	79% → 91%	2/3 sub-phenotypes performed better at validation site
Asthma/chronic obstructive pulmonary disease overlap	1–2	300	90% → 91%	38% → 54%	Although overall improved, performed worse at validation site possibly due to how the ML model used counts of features
Familial hypercholesterolemia	1–4	150	96–98% → 74–96%	N/A	Negative predictive value decreased
Atopic dermatitis	1–3	150	73–79% → 72–84%	51–54% → 63–75%	Mixed results across sub-phenotypes & sites

The “People Involved” column lists the estimated number or range of full-time equivalent persons involved with all aspects of the implementation, and includes programmers, clinicians, and computational linguists. Charts reviewed is the total number of patients’ charts reviewed for each phenotype, a sum of the charts reviewed for cases, and controls if applicable, at both the lead and validating sites. Precision and Recall columns list those statistics for the original computable phenotype rule-based algorithm vs. the new computable phenotype rule-based algorithm with NLP components added: arrows indicate change in these statistics from these original to new phenotype algorithms. Some algorithms have a range for precision or recall as either multiple (secondary) validation sites reviewed patients’ charts from which accuracy statistics were calculated, or there were separate precision/recall measures for sub-phenotypes. *N/A* not applicable: recall was not targeted for improvement in all phenotypes; thus, it was not calculated for all phenotypes.

Lastly, time to develop, and validate (including chart review) by lead and validation sites, was considerably longer than the subsequent implementation by other sites; specifically, 6 months or more for development and validation, versus only weeks for implementation. For example, ECG took 11 months to develop and validate, but sites only took anywhere from 1–3 weeks to implement. Also, sites reported that 1–2 servers were needed for executing the algorithms, although no further details were provided on server configuration.

### Themes

**Figure 1:**
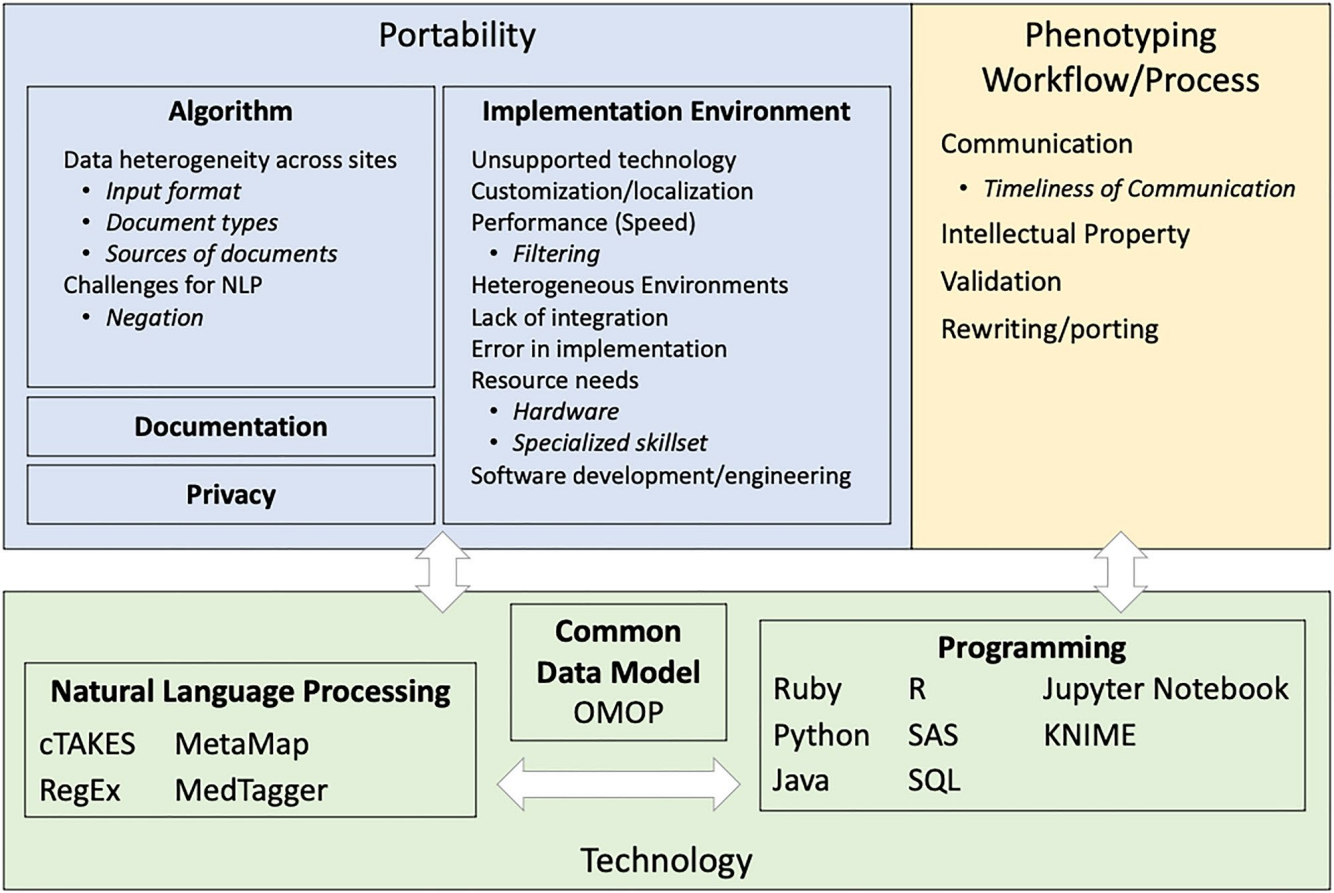
There were 3 overlapping themes (portability, phenotyping workflow/process, and (use of) technology. For each theme, sub-themes are shown in boxes with further sub-themes within each box listed as bullet points. For each lesson, if a technology was mentioned as being used, but there was no issue with the technology itself, the use of technology was simply noted. *NLP* natural language processing, *cTAKES* text analysis and knowledge extraction system.

Figure 1 shows the three major themes identified from the qualitative analysis: portability, phenotyping workflow/process, and technology. The technology theme was found to be a modifier for the other two primary themes, as all technologies were associated with another theme. This approach was used for the analysis and summarization phase to identify any recurring themes associated strongly with one or more technologies. Each of the themes is summarized in Table 3, with the full codebook available in Supplementary Appendix B.

**Table 3:** Summary of themes and challenges.

Theme	Challenges
Portability of algorithms	Algorithm performance varies by phenotype
	Identifying the correct type(s) of notes across sites can be challenging given differences how notes are categorized
	Well-known challenges in NLP and ML persist
Implementation environments	Use of different programming languages/NLP pipelines can cause delays in implementation when a site does not have local expertise
	Sites run NLP and ML in different environments, which may have different requirements for the software that can be run
	Local changes/customization were often needed for things like file paths and document input formats
	Data preparation steps were the most time and resource intensive
Privacy	Given identifiers embedded in clinical notes, sites have different requirements and restrictions on their use of notes for NLP
Documentation	Scripts and software often lacked sufficient documentation on how to execute, and the expected output
Phenotyping workflow/process	Communication delays between author and implementer could have compounding effects on overall time to complete
	Sharing NLP/ML pipelines with other sites may be hindered by intellectual property concerns
	Reconsider traditional workflows to phenotyping

Summary of the top themes found within our analysis, and a summary of the challenges reported by eMERGE sites within each theme. A full listing of themes is available in Supplementary Appendix B.

A few common sub-themes were identified, including both the portability and use of different technology. Filtering of data was also important, for both proper selection for the algorithm and appropriate filters to decrease the amount of data to improve performance of the software. Another important sub-theme was the need for human resources, both the need for team members with specialized skills to assist with the portability of technology, and the need for team members to communicate well.

### Summary of major challenges

#### Portability of algorithms

Considerations regarding the portability of phenotype algorithms were split into two sub-themes. The first theme was algorithm portability: how the ML and/or NLP algorithm performed at sites other than the lead site. This reinforced established observations that algorithm performance can differ by phenotype. For example, for atopic dermatitis, at a (secondary) validation site, many of the relevant dermatology records were captured on paper, from which text was not converted into parse-able format into the EHR, thus, the EHR-based algorithm had a high number of false negative results.

The format, composition, and classification of documents at different sites also played a role in algorithm portability, including the formats of clinical notes. This was an issue across all the types of notes used, which included ECG and other procedure and lab reports, and office/clinic encounter/visit notes. More often, sites described challenges in identifying the right documents to process with NLP. For example, the phenotype algorithm would require “radiology notes”, but no a priori semantic grouping was readily available at each implementing site for identifying broad categories of notes such as imaging, pathology, and microbiology. Instead, sites needed to review and map local document types to the document types specified in the algorithm. Similar issues occurred for the medical specialties/departments with which notes were associated, as well as the specific sections within notes. Manual review was often needed to resolve these issues. An unexpected finding was that the inclusion of general patient educational material in clinical notes also negatively impacted performance at some sites.

Finally, sites acknowledged that well-known challenges within NLP and ML persisted. The most prevalent challenge was negation: the task of inferring from the context of a term or phrase when it was not present or true. We observed several NLP components suffer performance losses because the modules failed to correctly capture some negation instances, e.g. “atrial fibrillation/flutter is no longer present” was falsely identified as a case. Accurate detection of negation can be difficult regardless of the NLP technologies used^49^. In addition to negation, language usage and document formatting can vary by institution or even across specialties at the same institution, which affected NLP performance. One example was the use of the colon as a separator in the text, which was interpreted in some sites as a terminator and in others as the start of a list. Diagnostic uncertainty (when the text indicates that the diagnosis is unclear) and rare terms were linguistic features also noted as issues, although we note NLP solutions may not exist to alleviate the former.

#### Implementation environments

The second sub-theme identified regarding portability was centered around the execution of the algorithm code—specifically, making the NLP/ML software run. Although NLP was restricted to two systems (cTAKES and MetaMap), setting up and executing these systems in different computing environments (e.g. different operating systems) introduced challenges. In addition, there were no restrictions placed on the programming languages used for ML and rule-based components of the phenotype algorithm as a whole. Sites noted that certain programming languages (e.g. Ruby) were not widely used across institutions. For some institutions, this meant the language was not supported, and as such the algorithm code could not be run. For others, the language was not the preferred language, and local experts had to be found to assist in the execution. This surfaced two additional themes for “Resource needs”: dedicated server environments to run the NLP/ML, and specialized staff—most often someone with experience in NLP.

Regardless of how familiar sites were with an NLP system or programming language, they frequently needed to modify the algorithm code before it would run locally (“Customization/Localization”). These changes were typically minor, such as changing file paths in the code and document input formats. Other changes included separate pre-processing steps for the clinical text—a technical solution to general problems noted in the “Data heterogeneity across sites” sub-theme.

Another difference noted across sites was “Performance (Speed)” as it relates to both the total elapsed time to get NLP/ML to run and the actual execution time. Sites noted that data preparation steps were typically the most labor intensive, and there was wide variation in time needed across sites. Execution time varied with computational resources and volume of the textual information available. With memory intensive text processing, one site noted that an NLP algorithm deployed as a Jupyter notebook on a PC with limited resources took “ > 2 h to run”, in response to which the site extracted the Python code and deployed directly to the server with augmented memory and disk space. Filtering of notes was a prevalent performance related theme. Some NLP algorithms as deployed would process all clinical notes, which at some sites was not feasible because of the very large numbers of notes at those sites, which at least at 1 site, were over 1 million notes, even after filtering. To address this, sites applied filters either by pre-selecting patients for whom to process notes or narrowing down to the appropriate clinical note types to process. Pre-selection/filtering of patients was very broad, such as selecting all patients whom had any diagnosis code for, or related to, the given phenotype.

Sites also noted how the use of multiple technologies (“Heterogeneous environments”) impeded portability. As previously noted, depending on the technology, local specialists were needed. Finding and coordinating availability of those individuals increased the total elapsed implementation time at some sites. Across multiple technologies, or sometimes even when using the same technology, the algorithm was implemented as disjoint scripts or programs (“Lack of integration”). Sites noted that they would need to run each of these steps separately, which also lengthened the total implementation time.

Additional themes relating to the software implementation were also noted by sites, including lack of boundary condition checks that caused software to crash. This included things such as unexpected null/empty/misformatted input. These also increased delays in the implementation as time was required to troubleshoot and resolve the issue.

#### Privacy

Sites reported that because clinical notes often contain patient identifiers, additional measures had to be pursued to assure patient privacy. One site required additional approvals to access clinical notes to run NLP. Another site observed that by running the NLP locally and distributing the final output/results, they obviated the complications of having to share entire clinical notes with the algorithm author. Therefore, by only sharing the outputs, this allowed sites and the network to maintain de-identified data, while still providing a deeper search into the EHRs of each institution.

#### Documentation

A lack of documentation crossed both the technical and algorithm themes. Sites noted sufficient documentation and instructions were not always available on *how* to execute the phenotype algorithm. In addition, insufficient documentation about *what* was the intended function of an algorithm, or the exact input needed, complicated the implementation. For the latter, sites would sometimes need to read the code itself, which also lacked sufficient documentation and/or comments.

#### Phenotyping workflow/process

During the implementation process, sites noted there were delays related to communication issues. For example, lack of documentation would prompt a site to request more information. While awaiting a reply, a site may have been required to shift focus away from the phenotype algorithm to another project, causing another delay before the site could shift focus back.

One site noted delays in implementation and dissemination due to intellectual property (IP) concerns at their institution. Since NLP and ML typically require significant investments in resources, an internally-developed system at this site was considered protected IP. The site worked to establish a version of the NLP algorithm that could be shared across sites. The considerable amount of time required to conduct the review and secure approvals delayed the overall implementation timeline.

An adjustment to the phenotyping process also included porting/re-writing code, which took on two forms. The first was specific to this study and driven by the network’s decision to limit the NLP pipelines that would be used. One site had a pre-existing NLP pipeline that was not one of the ones chosen; as a result, the site was required to port the NLP algorithm to cTAKES. Issues were identified in the ported version of the algorithm, which required correction. The second form of porting was driven by site-specific needs, requirements, or preferences to refactor or rewrite the provided algorithm. For example, one site rewrote a Ruby RegEx implementation in Python.

**Figure 2:**
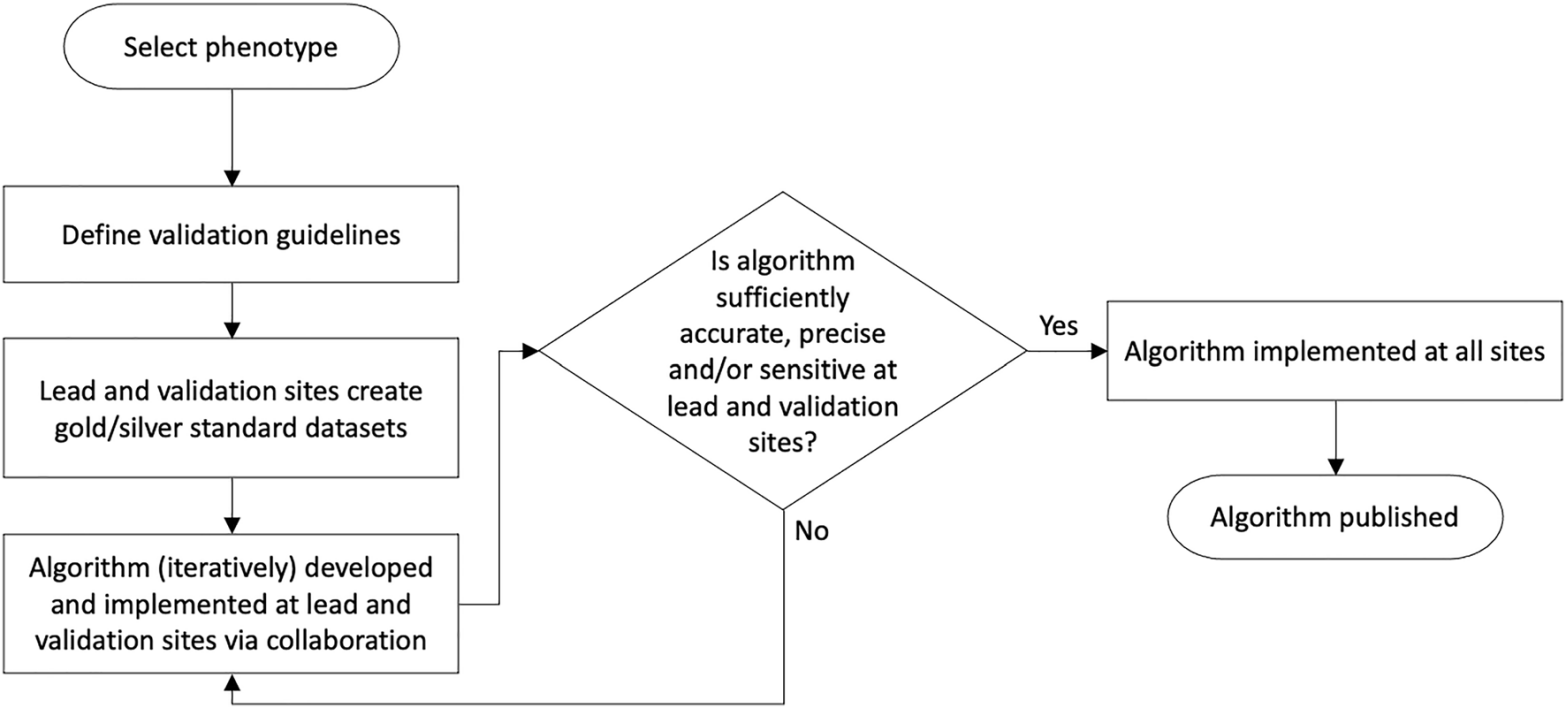
Flow diagram of proposed workflow for development, validation, and implementation of portable computable phenotype algorithms within eMERGE. The proposed workflow was adapted from a previously published workflow by Newton et al. on behalf of eMERGE^23^.

Overall, the network identified the need for and proposed a new phenotyping workflow to guide development and improve the process of validation (Fig. 2), especially for, but not limited to, NLP/ML algorithms. In the pre-existing workflow^23^, secondary site validation of algorithms did not commence until after a lead (primary) site develops and subsequently validates an algorithm. Therefore, the first workflow improvement is development of an algorithm at the lead site to be performed in parallel with the creation of a “gold standard” validation cohort by medical record review at both the lead and (secondary) validation sites, especially for NLP/ML algorithms that need training sets in order to develop the algorithm. This requires screening the EHR at the start of the workflow for a defined cohort from which the training and validation sets of patients are chosen. For example, a screen could be at least one International Classification of Diseases (ICD)-9/ICD-10 code for that phenotype as a highly sensitive filter. Consequently, selection of a random sample from a population enriched for that phenotype facilitates reasonable prevalence, usually in the 20–80% range. From this process, each site can select a random sample of perhaps 100–200 patients that clinicians classify as positive or negative cases, or undetermined, for a goal of at least 50 confirmed cases in each gold standard dataset. The algorithm developed in the primary dataset can be tested in the secondary dataset; therefore, if performance metrics are poor, the algorithm can be revised and tested in both sites’ datasets, without additional medical record review. Thus, the lead (phenotype creation) sites will encounter less inherent pressure to produce a “perfect” algorithm as a prerequisite to release to (secondary) validation sites, expediting the algorithm development process.

## Discussion

We leveraged the unique resources of the eMERGE network to assess the advantages and challenges of integrating NLP into portable computational phenotypes. Advantages of NLP include: improvement of sensitivity (SLE and ACO) for identifying more cases of a rarer condition; increased precision (CRS), an important consideration in more common conditions; and enabling deep phenotyping, such as extracting subphenotypes from ECG notes. In general, algorithm performance at both lead and validation sites was enhanced with the addition of portable NLP. Similarly, an implementation of a portable and computable phenotyping algorithm, for identifying patients for clinic trial recruitment, added NLP to their algorithm, improving algorithm recall and precision^26^.

NLP performance may vary between sites, due to heterogeneity in clinical document names and the basic structure of clinical notes. Ideally, the implementation of standardized terminology (e.g. LOINC Document Ontology) across all sites could provide explicit input descriptions and reduce inconsistency^18^. However, implementation of these standard terminologies is impractical due to the absence of clear selection criteria currently. The overall process could be costly, time-consuming, and difficult to change when insufficient evidence is available to guide the selection. Furthermore, even if all sites adopt the same terminology and CDM for the clinical notes, because the notes may vary in their local templates, documentation patterns, document quality (i.e. spelling mistakes and typos), overall EHR data quality, and sublanguages; portability is still challenging^16,26^. Thus, we suggest starting with semi-structured clinical notes (e.g. problem/medication lists): for example, recent studies have demonstrated the benefits of using allergy lists for clinical studies^50,51^.

Notably, the generalizability of negation modules remains an open NLP challenge, and is consistent with other reports^49,52^. Local tailoring on negation may be necessary, such as adding correction rules to the code for negating language. In addition, errors in the software code was another potential source of differing algorithm performance between sites. The use of formal collaborative version control systems (such as GitHub) should be prioritized over other less effective means such as e-mail distributions of code and documentation. For this and other reasons already mentioned, portability can be further improved by requiring institutions to improve development processes, provide comprehensive documentation, and customization options.

Successfully sharing and implementing a computable phenotype using NLP is not just about the NLP technology or the algorithm itself. Other critical factors include privacy protection, technical infrastructure setup, intellectual property agreement, and efficient communication. For example, as clinical notes are not always able to be de-identified, sites may be unable to exchange example notes, causing difficulties for cross-site validation. Recent advances on the Privacy-Protective Generative Adversarial Network may generate fake text data with retained structure similarity that can be used for NLP algorithm development and validation^53^. Federated learning approaches have also emerged to preserve privacy without needing to transport clinical text^54^. Formatting information embedded in notes (e.g. Rich Text Format [RTF]) has been shown to improve phenotyping results^55^; however, cross-site utilization of format information is used inconsistently across the eMERGE network. Infrastructure challenges may be ameliorated by cloud computing in which algorithms and data workflows can be prepackaged and used by researchers with little training^55,56^; however, institutions may not be comfortable putting protected health information (PHI) into a sharable cloud. Although not explicitly tested in this work, we also believe full-text indexing of all clinical notes at the beginning would speed up execution time and reduce infrastructure needs by narrowing down the notes to process with a rule-based NLP system.

Lastly, efficient and effective communication across sites is critical. Our traditional approach (i.e. communication via comments on PheKB.org), may be unsuitable for timely, iterative, bi-directional communication. Furthermore, as others have also noted, collaboration between sites and also between the different types of experts (i.e. clinicians, informaticists, etc.) needed is critical^23,27,29^. Additionally, developing a “simplicity metric” to characterize phenotyping algorithms would allow researchers to more easily determine the skills needed for implementation. For example, data types required by the algorithm could be ranked in order of simplicity of extraction from the EHR.

There are a few limitations to this study. First, the comparison of the performance using NLP pipelines other than MetaMap or cTAKES, such as CLAMP, was beyond our resources and timeline. Our approach to NLP platform selection was based on those with which we had the most experience, which is not necessarily based on the strengths or capabilities of the platform itself. While the advantage of our approach is that the results are likely more generalizable to organizations wanting to implement NLP enhanced phenotyping, sometimes by clinicians with minimal NLP training; the disadvantage is that it precluded using the most up to date NLP approaches, which could impact the results. A separate study may be needed to evaluate other pipelines’ performance. In addition, we were not able to assess how portable NLP performs for rare phenotypes: although we intended to identify patients with Brugada syndrome from ECG reports, we did not find sufficient cases for evaluation. As stated previously, sites were only asked to qualitatively evaluate their experiences, and gather quantitative data beyond performance statistics, in the last quarter of the 1 year pilot project; thus, sites had to at least partly rely on their memories, resulting in loss of some details. For example, unfortunately as significant time had passed, we could not accurately estimate hours spent; however, we felt it more important to report real time elapsed given the additional complexity noted needing to wait for team members across multiple sites to be available.In addtion, no formal, standardized measurement of time and effort was used, leading to reliance on estimates that could also lead to inconsistent reporting and inaccuracies. Finally, the number of charts reviewed for some of the phenotypes was small, and, for at least one phenotype, only 1 person reviewed the chart.

## Conclusion

In conclusion, incorporating NLP and ML into EHR phenotyping algorithms can improve phenotyping performance and enable deep phenotyping. Furthermore, while applying NLP at multiple sites entails several challenges, it is feasible to develop and implement phenotype algorithms with NLP/ML components with reproducible performance. Lastly, NLP requires dedicated personnel who are skilled in EHR phenotyping and NLP, and who communicate well. Given the value of mixed-methods evaluation of the portability of phenotype algorithms with NLP/ML, we recommend its use in studies of this type. While portable and replicable phenotype definitions and algorithms are possible, careful planning and architecture of the algorithms that support local customizations are expected to be needed for the foreseeable future.

## Supplementary Information

Supplementary Information.

## Data Availability

The data used for this work was from electronic health records which include identifiable data and thus cannot be shared per the HIPAA Privacy Rule. The code is available on PheKB.org under the page for each phenotype and survey data can be de-identified and available upon request by contacting the corresponding author, Jennifer A. Pacheco.
