# Supplementary material for: Evaluation of the portability of computable phenotypes with natural language processing in the eMERGE network
Source: Sci Rep. 2023 Feb 3;13:1971. doi: 10.1038/s41598-023-27481-y (PMC9898520; doi:10.1038/s41598-023-27481-y)
Supplement: Supplementary file 1 — Supplementary Information. [file 41598_2023_27481_MOESM1_ESM.docx]

**Appendix A – Survey Questions**

1. primary findings esp. changes in precision (PPV) & recall (sensitivity)
2. # of months to develop &/or validate &/or implement
3. resources (# people, servers, DBs, & any other resources) & issues w/ resources needed
4. # of subjects reviewed if validated, &/or # of cases &/or controls, & # of notes/records needed for implementation
5. pipeline (cTAKES, Metamap) &/or code (Regular Expression (RegEx) in Python, R, etc.) lessons inc. how long pipeline/code takes to get running & then to actually run
6. Portability, inc. any customization of alg. or code, or filtering of notes, ease of mapping document/note types/structure/sources across sites, etc.
7. Differences in previous algorithm without ML/NLP vs. updated/new alg. w/ ML/NLP
8. Any other ML/NLP specific issues, such as need for negation, difficulty extracting certain concepts, etc.
9. Any other technical issues such as data format, etc.
10. Any other barriers or lessons learned

**Appendix B – Codebook**

| **Code** | **Theme category** | **Theme level 1** | **Theme level 2** | **Theme level 3** | ***Description*** | **Summary of Challenges** |
| --- | --- | --- | --- | --- | --- | --- |
| **A** | **Portability** |  |  |  | *Any general concepts related to portability across institutions* |  |
| **A.1** |  | **Algorithm** |  |  | *Portability of the NLP/ML algorithm across the institutions - will capture more general aspects of how the algorithm works across institutions not captured by more specific codes.* | - Phenotype algorithms performed differently for different concepts, sub-phenotypes, and/or at different sites |
| **A.1.1** |  |  | **Data heterogeneity across sites** |  | *Differences in the data (including the notes). This may include not just differences in the content/writing styles of notes, but also attributes and features of data overall that relate to NLP or ML.* | - Rare diseases/observations may not be equally represented across sites - Unexpected note text (e.g., patient education material) can hamper NLP performance |
| A.1.1.1 |  |  |  | Input format | *How the data (including notes) have to be formatted to be input for use within the NLP/ML algorithm.* | - Conversion between native EHR document format was needed to make NLP run (e.g., RTF to text, multi-line to single-line documents). |
| A.1.1.2 |  |  |  | Document types | *Classification scheme of the documents themselves, including document sections.* | - NLP often requires constraining to certain categories of notes - Site naming of document types and document sections differs widely |
| A.1.1.3 |  |  |  | Sources of documents | *Who wrote the document; what department it came from; any considerations for portability with respect to the document source being a factor.* | - NLP often requires constraining to notes authored by certain specialties or in certain departments - Site names and availability of certain departments and specialties differs widely |
| **A.1.2** |  |  | **Challenges for NLP** |  | *Ongoing challenges that NLP as a field is still working to address; limitations for the specific implementation given constraints of text, time to implement, etc.* | - Even with many advances, challenges of applying NLP remain - Complications include rare terms, diagnostic uncertainty, formatting (e.g., colon as separator) - NLP/ML does not always improve performance |
| A.1.2.1 |  |  |  | Negation | *Specific issues regarding negation within NLP* | - Negation remains a particularly difficult problem for NLP |
| **A.2** |  | **Implementation Environment** |  |  | *Portability of the implementation - this will relate more to the software code/scripts/systems used to implement the specific NLP/ML algorithm. Considers what needs to be done to make software run.* |  |
| A.2.1 |  |  | Unsupported technology |  | *Technology that is not supported (including lack of familiarity with) at an institution* | - Institutions don't support or have experience with specific programming languages (e.g., Ruby) |
| A.2.2 |  |  | Customization/ localization |  | *Describes the local changes that were needed to get the technology to work at all, or to work in an optimized way.* | - Almost all implementations needed some local changes, but typically were minor - Customizations typically included file paths and document input formats |
| **A.2.3** |  |  | **Performance (Speed)** |  | *Elapsed time to develop, implement, and/or execute the NLP/ML software* | - Data preparation steps required the most human effort |
| A.2.3.1 |  |  |  | Filtering | *The need to filter notes in order to select the correct notes for NLP* | - Processing "all" notes is not always possible |
| A.2.4 |  |  | Heterogeneous environments |  | *Multiple technologies and/or environments used in conjunction for a single phenotype algorithm.* | - Multiple programming languages may require multiple implementers to be involved - Coordinating multiple implementers across teams can increase implementation time |
| A.2.5 |  |  | Lack of integration |  | *Multiple components (e.g., pipelines, scripts, other software) that are needed for the same phenotype algorithm but are not integrated in a cohesive manner.* | - Multiple scripts/programs were needed but not integrated or consistently documented - Running disparate scripts/programs increased implementation time |
| A.2.6 |  |  | Error in implementation |  | *Any technical errors (e.g., bugs, crashes) encountered during development and implementation* | - Software issues such as errors/crashing when not handling boundary conditions or empty input - Inconsistencies in script implementation by the same author over time |
| **A.2.7** |  |  | **Resource needs** |  | *Describes the resources needed to make the system run* |  |
| A.2.7.1 |  |  |  | Hardware | *Specific hardware requirements/needs. This can include cloud infrastructure as well as physical hardware* | - NLP/ML required more robust server environments to run - Certain technologies may have required dedicated environments at an institution |
| A.2.7.2 |  |  |  | Specialized skillset | *Skillsets in a specific area that were needed to execute the NLP/ML system at an institution* | - NLP/ML require a specialized skillset to be implemented at sites - Coordinating multiple implementers across teams can increase implementation time |
| A.2.8 |  |  | Software development/ engineering |  | *Considerations (not captured by other codes) that relate to general software engineering and development practices needed to create or that could be used to improve portability and implementation.* | - Code may be poorly modularized, difficult to read and interpret, or may lack documentation to run - Lack of centralized source control made software updates challenging |
| **A.3** |  | **Privacy** |  |  | *Privacy requirements for notes and other data* | - Clinical notes have additional privacy considerations and are not always available in a de-identified format - Additional permissions may be needed for an implementer to access clinical notes |
| **A.4** |  | **Documentation** |  |  | *Considerations or need for documentation in order for an institution to use/implement/configure the NLP/ML algorithm* | - Lack of documentation on an algorithm’s intent, processing assumptions, and requirements - Lack of documentation on assumptions regarding clinical note types |
| **B** | **Phenotyping Workflow/ Process** |  |  |  | *General process considerations; part of the phenotyping workflow* | - Large gaps in time between development efforts or from development to validation can hinder progress |
| **B.1** |  | **Communication** |  |  | *General communication that occurs within the phenotyping workflow* | - Timely, iterative, bi-directional communication is needed |
| B.1.2 |  |  | Timeliness of communication |  | *The process of porting from one technology to another* | - Implementers waiting on feedback from an author caused delays in the implementation process |
| **B.2** |  | **Intellectual property** |  |  | *Intellectual property needs in order to share/disseminate a phenotype algorithm* | - NLP/ML algorithms may be considered protected IP by organizations, precluding their sharing |
| **B.3** |  | **Validation** |  |  | *Validation process (including chart review)* | - Validation is also a localized process, differing by who is available to conduct validation - Validation needs may not always be clearly documented or communicated |
| **B.4** |  | **Re-writing / porting** |  |  |  | - Requiring authors to use a specific pipeline required porting existing, validated NLP pipelines - Lack of support for a specific technology required implementers to port a validated NLP pipeline |
|  |  |  |  |  |  |  |
| **C** | **Technology** |  |  |  | *Mentions of specific technologies and systems; this is planned to be used in conjunction with other codes. We are not interested in coding every mention of a specific NLP system or programming language.* |  |
| **C.1** |  | **NLP** |  |  |  |  |
| C.1.1 |  |  | cTAKES |  |  |  |
| C.1.2 |  |  | RegEx |  |  |  |
| C.1.3 |  |  | MetaMap |  |  |  |
| C.1.4 |  |  | MedTagger |  |  |  |
| **C.2** |  | **Programming** |  |  |  |  |
| C.2.1 |  |  | Ruby |  |  |  |
| C.2.2 |  |  | Jupyter Notebook |  | *Although Jupyter can include Python, R, etc., this will be used to capture where just Jupyter Notebooks are specifically mentioned.* |  |
| C.2.3 |  |  | Python |  |  |  |
| C.2.4 |  |  | R |  |  |  |
| C.2.5 |  |  | KNIME |  |  |  |
| C.2.6 |  |  | SAS |  |  |  |
| C.2.7 |  |  | SQL |  |  |  |
| C.2.8 |  |  | Java |  |  |  |
| **C.3** |  | **CDM** |  |  |  |  |
| C.3.1 |  |  | OMOP |  |  |  |
